# Telemedizin in der mobilen geriatrischen Rehabilitation: Ergebnisse der VITAAL-Studie

**DOI:** 10.1007/s00391-021-01987-4

**Published:** 2021-11-19

**Authors:** Sandra Burdinski, Susan Smeaton, Stefan Z. Lutz, Anja Partheymüller, Ugur Geyik, Gerhard W. Eschweiler, Florian G. Metzger

**Affiliations:** 1 Klinik für Psychiatrie und Psychotherapie und Geriatrisches Zentrum, Universitätsklinikum Tübingen, Calwer Str. 14, 72076 Tübingen, Deutschland; 2 Innovationszentrum Evangelische Heimstiftung Stuttgart, Stuttgart, Deutschland; 3 Rehabilitationsklinik Bad Sebastiansweiler, Mössingen, Deutschland; 4 Zentrum für Telemedizin, Bad Kissingen, Deutschland; 5 Institut für physikalische und theoretische Chemie, Universität Tübingen, Tübingen, Deutschland; 6 Vitos Klinik für Psychiatrie und Psychotherapie Haina, Haina, Deutschland

**Keywords:** Videovisite, Innovative Versorgungsmodelle, Qualitatives Design, Aufsuchende Rehabilitation, Digitalisierung, Innovative treatment models, Video visits, Qualitative design, Mobile geriatric rehabilitation, Digitalization

## Abstract

**Hintergrund:**

Um das Prinzip „Reha vor Pflege“ umzusetzen, bedarf es adaptiver Konzepte für geriatrische Patienten. Patienten mit Sehbehinderungen, eingeschränkter Kommunikationsfähigkeit, psychischen Erkrankungen oder kognitiven Defiziten sind in einer Rehabilitationsklinik oft nicht oder nur unzureichend behandelbar. Die mobile geriatrische Rehabilitation (MoGeRe) schließt diese Lücke im Versorgungssystem, ist aber in ihrer Reichweite begrenzt. Über die 22 Standorte in Deutschland ist es bislang nicht ansatzweise möglich, eine flächendeckende MoGeRe zu ermöglichen. Hier bietet die Telemedizin Lösungsmöglichkeiten.

**Fragestellung:**

Telemedizinische Ergänzungen der MoGeRe in Form von Videovisite und Videoaufnahme wurden hinsichtlich ihrer Machbarkeit und Akzeptanz in einer hochaltrigen Zielgruppe untersucht.

**Methode:**

Mit 25 Patienten wurden 101 Videovisiten und 26 diagnostische Videoaufnahmen durchgeführt. Interviews von Patienten und Teammitgliedern wurden mithilfe einer qualitativen Inhaltsanalyse ausgewertet.

**Ergebnisse und Diskussion:**

Insbesondere die Akzeptanz der Videovisite war bei allen Beteiligten hoch. Ihr Potenzial liegt in der Anpassung der individuellen Behandlung, Motivation, ärztlichen Lenkung sowie Supervision des Teams. Die Videoaufnahme kann die Chance bieten, den interdisziplinären Austausch zu bereichern und das therapeutische Prozedere zu evaluieren und anzupassen. Spezifische Strategien wie Begleitung von Angehörigen, Erklären des Prozedere und günstiges Timing sind bei kognitiv beeinträchtigten Patienten notwendig. Unsere Ergebnisse sind ein weiterer Beleg dafür, dass auch ältere Menschen als Nutzer digitaler Medien zu berücksichtigen sind.

## Abstract (en)

**Background:**

In order to implement the principle of rehabilitation before care, adaptive concepts for geriatric patients are required. Patients with visual impairments, impaired communication skills, mental illnesses or cognitive deficits are often not or only insufficiently treatable in a rehabilitation clinic. Mobile geriatric rehabilitation (MoGeRe) closes this gap in the care system, but its scope is limited. With the 22 locations in Germany, it is not possible to make a comprehensive MoGeRe possible so far. Telemedicine offers solutions here.

**Objective:**

Telemedicine supplements to MoGeRe in the form of video visits and video recording were examined with respect to their feasibility and acceptance in a very old target group.

**Method:**

A total of 101 video visits and 26 diagnostic video recordings were carried out with 25 patients. Interviews with patients and team members were evaluated with the help of a qualitative content analysis.

**Results and discussion:**

In particular, the acceptance of the video visit was high among all those involved. Its potential lies in the adaptation of the individual treatment, motivation, medical guidance and supervision of the team. The video recording can offer the opportunity to enrich the interdisciplinary exchange and to evaluate and adapt the therapeutic procedure. Specific strategies such as accompanying relatives, explaining the procedure and good timing are necessary for cognitively impaired patients. Our results prove that older people should also be taken into account as users of digital media.

Bei einer Vielzahl von hochaltrigen, multimorbiden Menschen kann von einem Bedarf an Rehabilitation ausgegangen werden, die die Lebenssituation potenziell erheblich verbessern könnte. So fanden Janßen et al. 2018 in einer Studie bei Menschen in der stationären Pflege einen allgemeinen Rehabilitationsbedarf bei mehr als jedem fünften Bewohner [5]. Die Behandelbarkeit dieser Patientengruppe in einer Rehabilitationsklinik ist jedoch nicht selten aufgrund von Defiziten wie hochgradiger Sehbehinderung, eingeschränkter Kommunikationsfähigkeit, psychischen Erkrankungen oder kognitiven Defiziten eingeschränkt. Dazu kommt, dass eine Behandlung in der Häuslichkeit Probleme zu lösen vermag, die im stationären Setting nicht zu lösen sind oder unter Umständen nicht einmal bewusst werden. Ein Beispiel ist das Wiedererlernen des Steigens von 3 niedrigen behindertengerechten Stufen in der Rehabilitationsklinik, während in der Häuslichkeit 10 steile Stufen überwunden werden müssen.

Unter dem Konzept der mobilen geriatrischen Rehabilitation (MoGeRe) wird eine aufsuchende Rehabilitationsbehandlung verstanden, die der Intensität einer stationären Rehabilitationsbehandlung entspricht. Diese Behandlung in der Häuslichkeit der Patienten kann die oben genannte Lücke im Versorgungssystem schließen. Lange Anfahrtszeiten bei ohnehin schon begrenzten Ressourcen in dem geriatrischen Bereich begrenzen jedoch die Umsetzbarkeit dieses Konzeptes. Zudem ist eine MoGeRe aktuell nur an 22 Standorten in Deutschland, davon 3 in Baden-Württemberg möglich. Dem standen 2017 161 geriatrische Fachabteilungen in Rehabilitationseinrichtungen mit zusammengenommen 8163 Betten gegenüber [3]; hinzu kamen im Datenjahr 2015 noch 222.600 Patienten in einer geriatrischen frührehabilitativen Komplexbehandlung [4]. Aktuellere Zahlen liegen bislang nicht vor.

Eine Verbesserung des Angebots könnte hier die Digitalisierung schaffen, die beispielsweise durch Videokommunikation Distanzen überwinden und Zeit einsparen kann. Interesse und Kompetenz in Bezug auf diese Technologie werden jedoch überwiegend jüngeren Menschen zugeschrieben, während ältere Menschen eher mit Technikscheue assoziiert werden [1]. Entsprechend liegen bislang kaum Studien vor, die den Gebrauch von Telemedizin durch hochaltrige Menschen untersuchen, im Bereich der Rehabilitation sind uns keine Studien bekannt. Altersunabhängige Studien zu telemedizinischen Anwendungen im Bereich der Kardiologie, Neurologie und Orthopädie liefern erste Hinweise darauf, dass diese Technologien eine gute Ergänzung zu konventionellen Verfahren darstellen. Im Bereich der Kardiologie konnte beispielsweise gezeigt werden, dass Telemonitoring das Mortalitätsrisiko reduziert [10]. In dem Bereich mit der häufigsten Anwendung von Telerehabilitation, der orthopädischen Rehabilitation, wurde für bestimmte Subgruppen gezeigt, dass eine ergänzende Telerehabilitation den Effekt einer Behandlung erhöht [2].

Mit dem Projekt VITAAL – Videovisite, Telemedizinische Applikationen und Alltags-unterstützende Assistenzsysteme in der Mobilen Geriatrischen Rehabilitation und Prävention – wurde eine telemedizinisch gestützte mobile geriatrische Rehabilitation auf ihre Machbarkeit hin untersucht. Das primäre Interesse dieser Studie galt neben der Umsetzbarkeit der telemedizinischen Komponenten in Form von Videovisiten und Videoaufnahmen der Akzeptanz der Technologie durch die hochaltrige Zielgruppe. Perspektivisch könnten telemedizinische Komponenten eine Erhöhung der Anzahl von Patienten ermöglichen, die diese Form der Behandlung in Anspruch nehmen können.

## Studiendesign und Methode

Die im Landkreis Tübingen ansässige Rehabilitationsklinik Bad Sebastiansweiler bietet bereits eine MoGeRe durch ein erfahrenes multiprofessionelles Team an. Die Aufnahme in die Behandlung ist bei Patienten mit einer Anfahrtszeit bis zu 20 min möglich.

Die Studie fand zwischen Mai 2019 und Oktober 2020 mit einer zweimonatigen coronabedingten Pausierung statt.

Die VITAAL-Studie wurde als Machbarkeitsstudie konzipiert. Die Patienten erhielten neben der MoGeRe, die üblicherweise 5 bis 7 Wochen mit 3 Behandlungstagen/Woche mit je 2 bis 3 Therapiesitzungen à 45 min umfasst, zusätzlich in der Regel einmal wöchentlich eine Videovisite mittels Tablets mit dem behandelnden Arzt. Die Durchführung fand in Anwesenheit eines Teammitglieds und im Bedarfsfall mit Angehörigen statt. Die Bedienung des Tablets und der Plattform „TeleView“, die eine Videokommunikation zwischen mehreren Personen ermöglicht, erfolgte durch das Teammitglied.

Zur besseren Verlaufsdokumentation wurden zudem im Bedarfsfall Videoaufzeichnungen von für die Rehabilitation relevanten Bewegungsfolgen wie beispielsweise das Gehen mit Hilfsmitteln oder feinmotorische Aktivitäten mit GoPro-Kameras aufgenommen.

Nach Beendigung der MoGeRe wurde mit den Patienten, und ggf. Angehörigen, ein leitfadengestütztes Interview zu den Themen Akzeptanz, Effekte und Adhärenz in Bezug auf die Videovisite und die Videoaufnahmen durchgeführt.

Mit dem therapeutischen Team wurden leitfadengestützte Fokusgruppen zu Vorerfahrungen, Akzeptanz, Effekten, Adhärenz und Potenzialen von Telemedizin vor Beginn der Studie, während des Verlaufs und nach deren Beendigung durchgeführt. Die Interviews wurden gemäß der qualitativen Inhaltsanalyse nach Mayring [7, 8] von 3 Wissenschaftlerinnen (S. S., L. N. und S. B.) ausgewertet. Eine qualitative Inhaltsanalyse ist ein hermeneutisches Verfahren zur inhaltlichen Textanalyse mit Anspruch auf Wissenschaftlichkeit.

## Stichprobe

25 Patienten, davon 17 Frauen und 8 Männer im Alter von 60 bis 93 Jahren (M = 81,6 Jahre) nahmen an der Studie teil. Ausschlusskriterien waren eine hochgradige Hörbeeinträchtigung und schwere Demenz, die die Durchführung einer Videovisite unmöglich machen.

Im Mini-Mental-Status-Test (MMST) wiesen 13 Patienten kognitive Defizite auf, davon eine Person mit leichter kognitiver Beeinträchtigung (25–27 P.), 9 im Sinne einer leichten Demenz (18–24 P.), eine im Sinne einer mittelschweren Demenz (10 bis 17 P.) und 2 Patienten im Sinne einer schweren Demenz (≤ 9 P.). Im Durchschnitt betrug der MMST 23,3/30 Punkte.

Vier Patienten lehnten das Interview im Verlauf ab, 2 Patienten waren aufgrund ihrer Demenz kommunikationsunfähig, und ein Interview mit den Angehörigen nicht möglich, bei 2 Patienten kam es zum Abbruch der MoGeRe, 3 Patienten verstarben während oder kurz nach der MoGeRe.

Mit 14 Patienten konnten die Interviews wie geplant durchgeführt werden.

## Ergebnisse

Für die Studie konnten 25 von 38 in diesem Zeitraum in die MoGeRe aufgenommenen Patienten als Probanden gewonnen werden.

Die Unterstützung der Angehörigen von kognitiv beeinträchtigten Patienten bei der Durchführung der Interviews erwies sich als erwartungsgemäß hilfreich bis maßgeblich. Acht von 25 Patienten erhielten eine entsprechende Begleitung bei dem Interview, überwiegend kamen sie in den Interviews jedoch selbst zu Wort.

Nach Analyse der Interviews wurden auf der Inhaltsebene in Übereinstimmung der 3 Analystinnen die Kategorien „Effekte“, „Beziehung“, „Technik“, „Akzeptanz“ und „Potenziale“ abgeleitet. Damit war ein hoher Bezug zu den Fragestellungen der Interviews gegeben.

Im Folgenden werden die Ergebnisse der qualitativen Inhaltsanalyse und Analyse der technischen Daten dargestellt.

Die Videovisiten und Videoaufnahmen waren technisch umsetzbar; auch in ländlichen Regionen und bei schlechtem Empfang bestanden Lösungsmöglichkeiten, um eine regelmäßige Kommunikation zwischen dem Patienten und dem Arzt zu ermöglichen. Eine ursprüngliche Skepsis im Team hinsichtlich der Technik und Akzeptanz durch die Patienten war nach wenigen Durchführungen nicht mehr vorhanden.

Innerhalb von 14 Monaten wurden mit den 25 Patienten 101 Videovisiten durchgeführt, von denen 9 aufgrund von technischen, personellen oder organisatorischen Gründen abgebrochen werden mussten. Die durchschnittliche Dauer betrug 7,3 min (SD ± 2,5 min). Die Bildfrequenz entsprach mit im Durchschnitt 25 „frames per second“ einem flüssigen Vollbild. Zusammengefasst wurde die Videovisite in Abwägung von Aufwand und Nutzen von dem gesamten Team als deutlich zugunsten des Nutzens bewertet.

Es wurden 26 Videoaufnahmen gemacht, von denen 11 Aufnahmen problematisch waren, im Sinne von Technik, Erschöpfung und dunklen Räumlichkeiten.

Die Akzeptanz der Videoaufnahmen war mit 15 von 25 Einverständniserklärungen bei den Patienten und deren Angehörigen geringer ausgeprägt als die der Videovisiten (25/25). Hier spielten v. a. aversive Emotionen gegenüber dem Gefilmtwerden eine Rolle. Ähnliche Ressentiments waren auch aufseiten von Behandlern vorhanden. Dem gegenüber stand die Wahrnehmung einer erhöhten Selbstreflexion im Vorfeld der Aufnahme, die sich in Kombination mit der interdisziplinären Supervision im Team als hilfreich und informativ für das therapeutische Procedere erwies. Der Aufwand im Zusammenhang mit den Videoaufnahmen erwies sich als deutlich höher als in Bezug auf die Videovisite (Mitführen und Aufbau der Geräte etc.) Eine abschließende Bewertung war bei fehlender Routine noch nicht möglich.

Hinsichtlich der Videovisiten wurde auf Patienten- und Teamseite eine hohe Akzeptanz als sinnvolles Instrument einer rehabilitativen Behandlung deutlich. Ohne Ausnahme äußerten sich alle Behandelnden nach der Anwendung von Videovisiten diesbezüglich positiv, ebenso eine Mehrheit von 8 Patienten. Voraussetzung dafür schien eine persönliche Note des Kontaktes zum Arzt zu sein, die die Videovisite in der Wahrnehmung der Patienten zu einer echten Alternative zum Präsenzkontakt macht. Als „ähnlich, aber anders“ fasste eine Patientin den Vergleich zwischen den Alternativen stellvertretend für die Mehrheit der Befragten zusammen. Eine Minderheit der Patienten empfand die räumliche Distanz zum Arzt als Fehlen der persönlichen Note und bevorzugte den Präsenzkontakt. Hier schien auch eine reine Gewöhnung an den Besuch einer Praxis eine signifikante Rolle zu spielen. Insgesamt wurde die Prägung der hochaltrigen Zielgruppe durch ein hausärztliches Arztbild und eine positive Bewertung des reinen Arztkontakts deutlich. Eine Relevanz der Videovisite für den Behandlungsverlauf wurde von einer knappen Mehrheit der Patienten verneint. Vor allem die selbstständigen und kognitiv unbeeinträchtigten Patienten bewerteten die Physiotherapie als einzig relevante Komponente und diese als nichtverbesserungsbedürftig durch den Kontakt zum Arzt. Sie nahmen davon ausgehend keinen Effekt der Videovisite wahr und benötigten selbstanamnestisch auch keine Motivation im Behandlungsverlauf. Patienten, die mehr Motivation, Beratung und Anpassung der Therapie bedurften, profitierten vermutlich entsprechend mehr von den Videovisiten.

In Bezug auf kognitive Defizite stieß die Videovisite bei mittelgradig bis schwer beeinträchtigten Patienten an Grenzen. Sie zeigten schneller Überforderung und Irritationen bis hin zur Verwirrtheit durch die für sie wiederholt nichtnachvollziehbare Maßnahme. Eine Erklärung der Videovisite musste daher in den Ablauf einer Sitzung eingeplant werden, um die Akzeptanz der Patienten zu sichern, Ängste abzubauen und Stress zu reduzieren. Die Begleitung von Angehörigen oder im Bedarfsfall Übernahme der Gespräche erwiesen sich als unverzichtbar, um eine sinnvolle Gestaltung zu ermöglichen und negative Effekte zu minimieren. Es erwies sich als hilfreich, die Videovisiten vor dem Beginn der therapeutischen Behandlung durchzuführen, um Nervosität zu mindern und die Konzentration im Anschluss der Videovisite ganz auf die therapeutische Behandlung fokussieren zu können.

Für das Behandlungsteam bot die Videovisite einen deutlichen Nutzen durch die kontinuierliche ärztliche Betreuung der Patienten, durch die wiederholte Motivation des Patienten und daneben zusätzlich durch einen erleichterten Zugang zu Angehörigengesprächen mit dem Potenzial einer weiteren Effektivitätssteigerung. Zudem erbrachte die Videovisite die Chance des kurzfristigen Anpassens individueller Behandlungsziele, die auch mehrfach genutzt wurde.

Eine bessere Supervision und Lenkung des therapeutischen Teams durch den Arzt wurden auch speziell durch die visuellen Informationen möglich, die durch andere Maßnahmen wie Dokumentationen, Berichte oder Telefonate mit Patienten nicht vorhanden sind. Auch wenn eine Bezifferung schwierig ist, wurde übereinstimmend von einer signifikanten Qualitätssteigerung ausgegangen. Ein Mehraufwand entstand im Hinblick auf die Videovisite v. a. durch den Organisationsaufwand und die Integration der Videovisiten in den ärztlichen Arbeitsalltag.

Das größte Potenzial sah das Behandlungsteam in einer Vernetzung der digitalen Dokumentation mit Integration der Videoaufnahmen in eine elektronische Patientenakte, die den zeitgleichen Zugang zu wesentlichen Informationen erleichtern würde. Das Potenzial der Videovisite liege primär in dem auch für in der Mobilität und Kognition eingeschränkte Patienten erleichterten Zugang zu einem ärztlichen Kontakt sowie für Patienten ohne relevante kognitive Defizite auch in Form einer selbstständig initiierten Videosprechstunde bei einem entsprechenden Angebot.

## Diskussion

Videovisite und Videoaufnahmen erwiesen sich als geeignete Instrumente zur telemedizinischen Ergänzung einer MoGeRe. Sie sind unter spezifischen Voraussetzungen wie Übernahme der Bedienung der Technik durch Mitglieder des therapeutischen Teams, empathischem Erklären der Indikation und günstiger Einbindung in den Therapieablauf auch bei hochaltrigen und kognitiv eingeschränkten Patienten einsetzbar und werden von den Patienten akzeptiert, insbesondere die Videovisite.

Das Ergebnis lässt sich durch das Modell Unified Theory of Acceptance and Use of Technology (UTAUT) von Venkatesh et al. [12] erklären, welches die Nutzung von Technologie anhand von vier Variablen voraussagt, nämlich dem erwarteten Nutzen, dem erwarteten Aufwand, sozialen Einflussfaktoren und erleichterten Bedingungen (im Sinne von Trainingsangeboten und Unterstützung). Es ist davon auszugehen, dass in Bezug auf die hochaltrige Zielgruppe besonders der geringe Aufwand und die angebotene Unterstützung die Akzeptanz und den vergleichsweisen hohen Anteil an Studienteilnehmern begünstigt haben.

Kongruent ist das Ergebnis mit einer Studie zu virtuellen Arztbesuchen in British Columbia, bei der 7286 virtuelle Arztbesuche bei 5441 Patienten bezüglich Inanspruchnahme durch verschiedene Altersgruppen und der Akzeptanz untersucht wurden. Die Studie zeigte eine höhere Inanspruchnahme durch jüngere Patienten; die geringste Inanspruchnahme war bei den ältesten Patienten (≥ 85 Jahre) gegeben. Die virtuellen Arztbesuche wurden von 93,2 % als hochqualitativ beurteilt, 91,2 % berichteten einen hohen oder mittelgradigen Nutzen in Bezug auf die Behebung ihrer Gesundheitsprobleme. Die Autoren schlussfolgern, dass virtuelle Arztbesuche ein Mittel sind, um das Gesundheitssystem patientenorientierter zu gestalten [9]. Die Autoren des aktuellen 8. Altersberichts des BMFSJ ergänzen, dass „mithilfe virtueller Arztbesuche die Patientenversorgung in benachteiligten Regionen verbessert wurde, dass diese Arztbesuche von einer substanziellen Zahl älterer Menschen angenommen wurde und für diese eine Ergänzung konventioneller Patientenversorgung bildet“ [1].

Ähnliche Daten lieferte auch eine Umfrage der University of Michigan National Poll on Healthy Aging von 2019, die 50- bis 80-Jährige zu Erfahrungen und Meinungen bezüglich Telemedizin befragte. Ausgehend von einer historisch begründeten Beziehung zwischen Patient und Arzt in Form eines Präsenzkontaktes deuten die Autoren diese Ergebnisse als Paradigmenwechsel, der durch Fortschritte der telemedizinischen Technologien ermöglicht wird und neue Möglichkeiten für ältere Menschen bietet [6].

### Einordnung des Projektes VITAAL in den aktuellen Stand der Digitalisierung in der Medizin

Die VITAAL-Studie stellt ein Projekt der Digitalisierung medizinischer Leistungen im Rahmen der medizinischen Regelversorgung am besonderen Bedarf der MoGeRe dar. Im Verlauf des Projektes ereignete sich die Coronapandemie, die in beinahe allen Bereichen des Lebens Veränderungen, oftmals im Sinne einer Digitalisierung, brachte. Ein Großteil der Daten wurde jedoch bereits vor der Pandemie erhoben. Aufgrund der während der Pandemie erhobenen nur geringen Daten und der pandemiebedingten Umstellung des Teams ist ein echter Vergleich vor und während der Pandemie nicht möglich. Schon vor der pandemiebedingten Pause von VITAAL trat eine Sättigung der qualitativen Daten ein. Trotz der pandemiebedingten gesellschaftlichen Veränderungen konnten nach der Pause wider Erwarten keine wesentlichen neuen und zusätzlichen Erkenntnisse aus der qualitativen Analyse gewonnen werden.

Letztlich ist aus der Situation heraus kein anderes Ergebnis bezüglich der (frühen) Pandemieauswirkungen auf VITAAL und damit auf die Digitalisierung der mobilen geriatrischen Rehabilitation erwartbar gewesen. Die durch Pandemie erheblich katalysierte Digitalisierung betraf und betrifft das Gesundheitswesen weniger als z. B. die Wirtschaft oder das Schulwesen. Im Gesundheitswesen haben sich die Digitalisierung und insbesondere die digitale Kommunikation stärker zwischen den professionell Tätigen entwickelt als zwischen den Therapeuten/Ärzten und den Patienten. Trotz der pandemiebedingt beschleunigten Digitalisierung ist die Arzt/Therapeut-Patient-Kommunikation deshalb weiterhin ein wichtiges Forschungsfeld. Wissenschaftliche Untersuchungen zur digitalen Arzt/Therapeut-Patient-Kommunikation sind im psychiatrisch-psychotherapeutischen Bereich bereits durchgeführt worden [11, 13], jedoch sind diese Ergebnisse mit dem hier untersuchten geriatrischen Bereich nicht vergleichbar, da es sich in aller Regel um jüngere, technikaffinere Patienten handelt, die nicht an einer geriatrietypischen Einschränkung der Kommunikationsfähigkeit leiden. Die Relevanz der aktuellen wissenschaftlichen Untersuchung dieser digitalisierten Arzt/Therapeut-Patient-Kommunikation bei älteren Patienten ist dementsprechend durch die bereits erfolgte pandemiebedingte Digitalisierung nicht vermindert, sondern eher erhöht, sodass in diesem Gebiet weiterhin ein hoher Forschungsbedarf besteht.

Die aktuelle Studie ist im Bereich der Rehabilitationsbehandlungen angesiedelt. Eine Übertragung von Erkenntnissen aus der Digitalisierung der ambulanten Versorgung in diese Versorgungsform ist nicht so leicht möglich, wie es zunächst erscheinen mag, da es sich um eine hybride Behandlungsform, mit verschiedenen Behandlern aus verschiedenen Berufsgruppen über einen begrenzten, festgelegten Zeitraum handelt.

### Schlussfolgerungen

Zusammenfassend lässt sich feststellen, dass der Megatrend Digitalisierung auch die heute hochaltrigen und sogar kognitiv beeinträchtigten Menschen einschließen könnte, sofern sie als autonome Personen dies wünschen. Mindestens in dem dargestellten Setting einer MoGeRe können die entsprechenden Voraussetzungen, inklusive Herstellens der technischen Voraussetzungen, Schulung und Motivation der Behandler und Anpassen der strukturellen Organisation, auch in absehbarer Zeit geleistet werden. Dann bietet sich die Chance, die individuelle Versorgung dieser Patientengruppe durch zeitgemäße Methoden zu verbessern oder in vielen Fällen erst zu ermöglichen.

## Fazit für die Praxis

- Videovisiten werden auch von diesbezüglich unerfahrenen älteren Menschen akzeptiert und wahrgenommen, sofern die Rahmenbedingungen stimmen.
- Die Rahmenbedingungen beinhalten Unterstützung bei oder Übernahme der Bedienung der Technik und eine ausreichende Ton- und Bildqualität.
- Besondere Strategien und erhöhter Zeitaufwand sind bei kognitiv beeinträchtigten Patienten notwendig.
- Eine konstante ärztliche Betreuung ermöglicht die wiederholte Motivation des Patienten, Beratung, Anpassung der Behandlung sowie bessere Lenkung und Supervision des therapeutischen Teams.
- Der Nutzen im Sinne einer Qualitätssteigerung von Videovisiten und Videoaufnahme überwiegt den Aufwand.
- Es besteht weiterer Forschungsbedarf, um eine Verbesserung der Versorgung geriatrischer Patienten zu ermöglichen.
